# Assessing Drinking Water Quality at the Point of Collection and within Household Storage Containers in the Hilly Rural Areas of Mid and Far-Western Nepal

**DOI:** 10.3390/ijerph17072172

**Published:** 2020-03-25

**Authors:** D. Daniel, Arnt Diener, Jack van de Vossenberg, Madan Bhatta, Sara J. Marks

**Affiliations:** 1 Department of Water Management, Delft University of Technology, 2628CD Delft, The Netherlands; 2 Department of Water Supply, Sanitation and Environmental Engineering, IHE Delft Institute for Water Education, 2611 AX Delft, The Netherlands; j.vandevossenberg@un-ihe.org; 3 Department of Sanitation, Water and Solid Waste for Development (Sandec), Swiss Federal Institute of Aquatic Science and Technology-Eawag, CH-8600 Dübendorf, Switzerland; arnt.diener@outlook.com (A.D.); sara.marks@eawag.ch (S.J.M.); 4 Helvetas Swiss Intercooperation Nepal, Jhamshikhel Dhobi Ghat, Lalitpur, GPO Box 688 Kathmandu, Nepal; Madan.Bhatta@helvetas.org

**Keywords:** water quality, *E. coli*, sanitary inspection, household hygiene, hilly area, rural communities, Nepal

## Abstract

Accurate assessments of drinking water quality, household hygenic practices, and the mindset of the consumers are critical for developing effective water intervention strategies. This paper presents a microbial quality assessment of 512 samples from household water storage containers and 167 samples from points of collection (POC) in remote rural communities in the hilly area of western Nepal. We found that 81% of the stored drinking water samples (mean log_10_ of all samples = 1.16 colony-forming units (CFU)/100 mL, standard deviation (SD) = 0.84) and 68% of the POC samples (mean log_10_ of all samples = 0.57 CFU/100 mL, SD = 0.86) had detectable *E. coli*. The quality of stored water was significantly correlated with the quality at the POC, with the majority (63%) of paired samples showing a deterioration in quality post-collection. Locally applied household water treatment (HWT) methods did not effectively improve microbial water quality. Among all household sanitary inspection questions, only the presence of livestock near the water storage container was significantly correlated with its microbial contamination. Households’ perceptions of their drinking water quality were mostly influenced by the water’s visual appearance, and these perceptions in general motivated their use of HWT. Improving water quality within the distribution network and promoting safer water handling practices are proposed to reduce the health risk due to consumption of contaminated water in this setting.

## 1. Introduction

Nearly half a million deaths per year are attributed to inadequate drinking water supplies [1]. Despite remarkable progress in extending access to improved water sources over the past decades, an estimated 2.2 billion people still rely on sources contaminated with fecal bacteria, the vast majority of whom live in rural areas of low- and middle-income countries (LMICs) [2]. Many rural households must fetch their daily water away from the home, presenting a further opportunity for recontamination during transport, storage and handling [3,4,5,6]. 

While many water-quality studies have been conducted in rural settings in LMICs, little research has been dedicated to characterizing water quality in the most remote locations, especially in alpine regions [7]. The lack of systematic water quality assessments in remote alpine areas can be explained by the challenges of conducting testing in such resource-contrained settings. Standard microbial assays are especially difficult under alpine conditions, since they are temperature sensitive, require access to reliable power, material supply chains, and considerable capital investments in equipment [8,9]. Moreover, alpine communities must contend with the geographic challenges of difficult road access and limited infrastructure development, both of which further complicate water-quality testing [10]. 

This study presents a cross-sectional analysis of microbial quality of drinking water across five rural communities in the hilly alpine area of western Nepal. This region is characterized by high elevation, seasonal rainfall, subsistence livelihoods, and limited road access [11]. The 2016 Nepal Demographic Health Survey (DHS) reported that 53% of people in rural Nepal had access to a piped water scheme and 21% practiced open defecation. The prevalence of diarrhea among children under five years was 8%. The risk of water-related disease was high because only 12% of rural inhabitants performed appropriate household water treatment (HWT) [12]. Additionally, people in the southern part of Nepal also suffer from exposure to high arsenic contamination in their drinking water [13,14].

The specific objectives of this study were: (1) to characterize microbial water quality at the location where households draw water, hereafter called “point of collection” or POC, and within household storage containers, (2) describe the relationship between microbial water quality at these two sampling points across the five villages, and (3) assess the potential for water treatment interventions based on households’ perceptions of their drinking water quality and current treatment practices. 

## 2. Method

### 2.1. Study Setting

**Figure 1:**
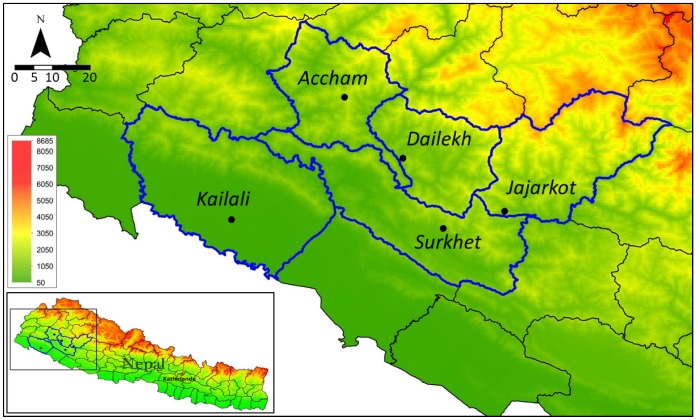
Location of the study area in Mid and Far-Western Nepal. Study villages are marked as black dots with district names shown.

Field data collection was conducted from October to December 2014 in five villages in five districts in Mid and Far-Western Nepal: Surkhet, Dailekh, Jajarkot, Accham, and Kailali. The first four districts are located in the hilly area (750–2000 m.a.s.l.), and Kailali is located in the Terai (<300 m.a.s.l.), a relatively flat area close to the border of India (Figure 1). 

The study was part of a baseline study initiated by Helvetas Swiss Intercooperation-Nepal and the Swiss Federal Institute of Aquatic Science and Technology (Eawag) to assess drinking water quality across Helvetas’ Integrated Water Resources Management (IWRM) program area. The five villages were selected since they were representative of the IWRM service area in the five districts, which were planned to receive water, sanitation, and hygiene (WASH) interventions after completion of the baseline study. 

Following village selection, household enrolment was based on a two-stage sampling method: first, three to five sub-villages were randomly selected within each village. Afterwards, households were randomly selected within the selected sub-villages along transects. Between 74 and 129 households were enrolled in each village, for a total of 512 households. A questionnaire that probed households’ perceptions and practices related to WASH was conducted, including a structured inspection of observable sanitary conditions. More details on the household interview can be found in Daniel et al. (2019) [15], which focuses on the behavioral aspects of household water treatment. Informed consent was obtained from all participants prior to the interview and from community or village leaders before the project. All activities planned through the Helvetas-Eawag research collaboration was approved by the Department of Water Supply and Sewerage (DWSS) in Nepal.

### 2.2. Water-Quality Testing

Briefly, the strategy involved a network of decentralized semi-permanent field laboratories with teams of sample collectors and laboratory technicians [10,16]. Six local enumerators were hired to conduct the interviews and structured observations at households and collect the water samples. The enumerators were chosen based on their a familiarity with the study area and experience in survey-based research. A training and pilot test for conducting interviews and collecting water samples took place before the data collection began. Immediately after the interview was finished, the enumerator asked for permission to collect a water sample from the household’s water storage container. The respondents were asked to fill a 100 mL Whirl-Pak bag (Nasco, Fort Atkinson, WI, USA) containing sodium thiosulfate to neutralize any chlorine residual in the sample. The enumerator recorded the household’s identification and distance to the POC (self-reported walking time in minutes), which, if available at the time of the visit, was also sampled. 

**Figure 2:**
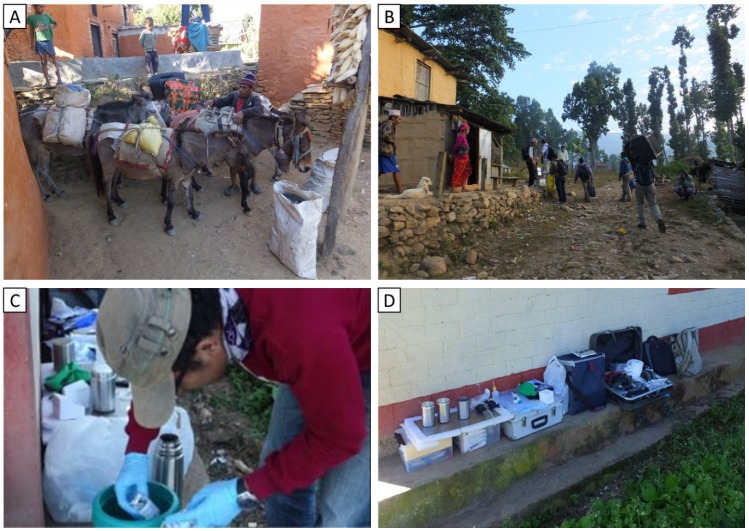
(**A**): Mules for transporting lab equipment. (**B**): Carrying equipment to a location without road access. (**C**): Thermos bottle to transport samples. (**D**): Adaptable field water testing kit including solar-powered incubation system.

The 100-mL water samples were placed inside a thermos bottle without ice during the transportation process to keep the temperature low, i.e., 10–15 °C. They were carried to the portable field lab that was established in each village (Figure 2) and processed within 6 h. The World Health Organization’s (WHO) procedure for microbial water testing by membrane filtration technique was followed: a 100-mL sample was filtered through a 0.45 µm pore size membrane. All the samples were incubated at 35 ± 2 °C for 24 h [17]. Positive and negative controls were processed daily, and triplication was undertaken every 20 samples for a total of 26 triplications. We performed the positive control by adding a small portion of animal feces into a water sample and the negative control using sterile water. More information about the field laboratories’ set up and operation is published elsewhere [10,18].

After incubation, the colony-forming units (CFU) of *E. coli* and total coliform that appeared on Compact Dry EC plates (CDP, HyServe GmbH and Co, Uffing, Germany) were counted and reported in concentration units (CFU/100 mL). According to the manufacturer’s recommendations, counts higher than 250 colonies were reported as too numerous to count (TNTC) [19]. For the present study, which is concerned with the health risks associated with faecal contamination of drinking water, the analysis focuses on *E. coli* as recommended by the WHO [17]. 

### 2.3. Data Analysis

The household interview and water quality data were compiled using Microsoft Excel and imported to IBM SPSS 24 for statistical analysis. *E. coli* concentration data were exponentially distributed, so these variables were log_10_ transformed, with all zero values being replaced by 0.5 prior to transformation. This approach is commonly used in environmental microbiology studies when analysing and reportings both mean and standard deviation; see for example [4,20,21]. However, since the transformed data did not meet the assumptions of parametric testing methods, i.e., assumptions of normality or linearity, we used non-parametric testing equivalents for the water quality-related analyses; see for example another study that used non-parametric tests for log transformed data [22]. The following non-parametric tests were used for all water quality-related analysis: (1) Kruskal–Wallis (with sign *H*), (2) Wilcoxon signed-rank (*Z*), Mann–Whitney U (*U*), and Spearman rank-order correlation (*r_s_*). To analyse the relationship between households’ socio-economic characteristics, water quality-related perceptions, and their use of HWT, the following tests were used: (1) Pearson correlation (*r*) for parametric analysis, (2) Spearman correlation (*r_s_*), and Chi-squared (*X^2^*) for categorical variables. 

Statistical results were reported in standard American Psychological Association (APA) format, e.g., Spearman rank correlation is reported in terms of (*r_s_* (df) = coefficient, p-value); where *r_s_* = the correlation coefficient, df = degrees of freedom, and p = probability value of obtaining at least the observed results. Differences between groups were considered to be statistically significant at a p-value ≤ 0.05. A relative wealth index was constructed from reported household assets using principal component analysis (PCA) [23] to analyse its potential association with households’ use of HWT. Detailed information on the wealth index can be found in [15]. 

## 3. Results

### 3.1. Household Characteristics

Table 1 shows the information on respondents’ characteristics. 59% of the respondents (n = 303) had been exposed to HWT promotional activities, i.e., promoting use of HWT methods and information regarding their benefits. From the 388 respondents who reported the distance to the main water source, 35% of them had their main water source within 5 min walking distance, 47% within 5 to 15 min walking distance, 11% within 15 to 30 min, and 7% more than 30 min. In terms of access to sanitation facilities, 90% of households had their own pour-flush pit latrine toilet, 6% used a shared or public toilet, and 4% practiced open defecation. About half of the households visited (n = 265) had at least one child under the age of five, with 5% of the children (n = 13) having experienced an episode of diarrhea in the previous two weeks. Almost all households (93%) had a home with an earthen floor, 28% had a roof made from mud or straw, and 9% had walls made of concrete. 

Enumerators directly observed the household setting (n = 512) to assess hygienic conditons. 32% of respondents did not use a lid to cover their water storage container. Almost all of the respondents (99%) reported cleaning their water storage container; a quarter of these households regularly washed their container with soap or chlorine, while the majority used only water. Additionally, among 419 respondents, 404 of them (96%) used different containers for transport and storage. Among the 200 stored water containers observed, 34% did not have livestock nearby (i.e., on the household plot) and 27% were free from the presence of flies.

### 3.2. Water Quality

**Figure 3:**
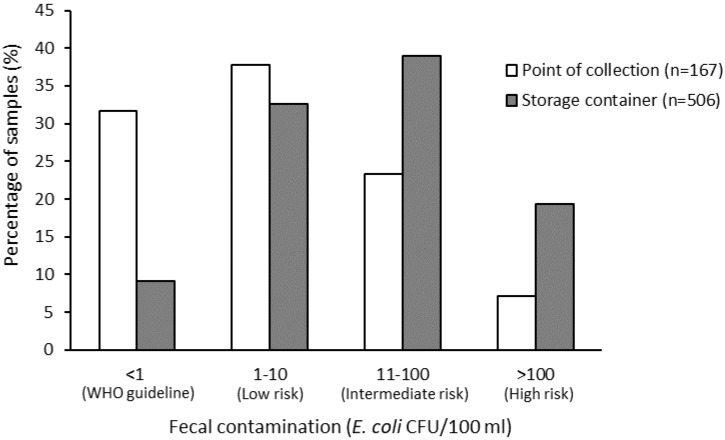
*E. coli* concentration at the point of collection (POC) and in households’ water storage container.

Overall, 32% of POC samples and 9% of household stored water samples were free from *E. coli* (Figure 3), with 58% of these stored water samples having *E. coli* concentrations at an intermediate or high risk level [24]. The mean log_10_ concentration of *E. coli* in household stored samples was 1.16 CFU/100 mL (SD = 0.84) compared to 0.57 CFU/100 mL (SD = 0.86) in POC samples. There was a negative correlation between the number of hours that water services were available per day and the log_10_ concentration of *E. coli* at the POC (*r_s_* (89) = −0.243, *p* = 0.022). 

**Figure 4:**
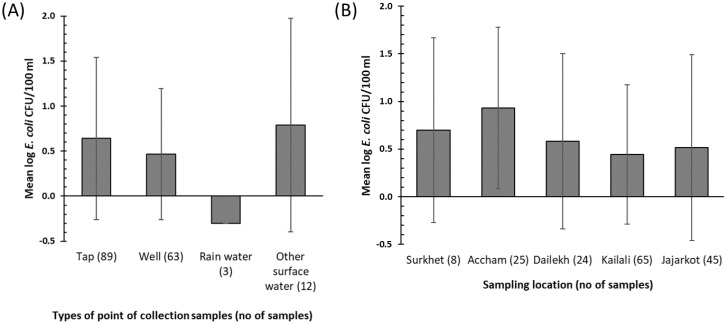
(**A**): Mean log_10_ *E. coli* concentration detected in the different types of POC (standard deviation bars shown). (**B**): Mean log_10_ *E. coli* concentration detected at the POC in each district. A measurement of zero *E. coli* was replaced by 0.5 to enable the log transformation.

Compared to other sources, surface water had the highest mean log_10_ *E. coli* concentration of 0.78 CFU/100 mL (SD = 1.18) (Figure 4A). Tube wells had a mean log_10_ *E. coli* concentration of 0.46 CFU/100 mL (SD = 0.73), with only rainwater having a lower average contamination level. All three samples collected from rainwater harvesting tanks had no detectable *E. coli* contamination. We found no significant difference in microbial water quality among POC source types (*H* (3) = 5.65, *p* = 0.13). Moreover, there was no significant difference between the water quality at the POC in different villages (*H* (4) = 6.56, *p* = 0.16) (Figure 4B). 

**Figure 5:**
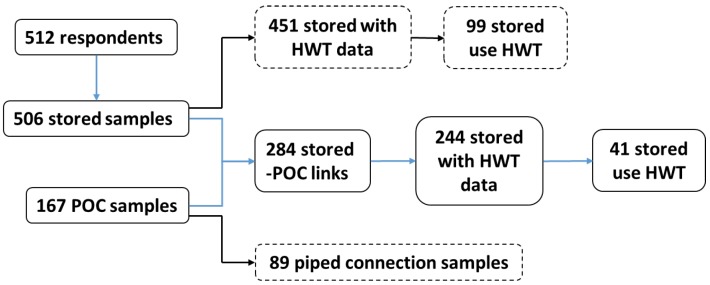
Schematic flow of the water-quality analysis showing the link between stored samples and POC samples, the number of POC samples coming from a piped connection, and the number of stored samples coming from treated water.

Out of 506 household-stored water samples, 284 were linked to 167 POC samples, indicating that some households shared the same POC (Figure 5). Among these paired samples, the mean log_10_ concentration of *E. coli* in stored water containers was 1.13 CFU/100 mL (SD = 0.87) compared to 0.70 CFU/100 mL (SD = 0.88) at the POC, and 180 stored water samples (63%) had a greater *E. coli* concentration than the POC. There was a significant increase in the concentration of *E. coli* from the POC to the stored water container (*E. coli* in *63*% of stored samples > POC; *Z* = −6.56, *p* < 0.001). 

**Figure 6:**
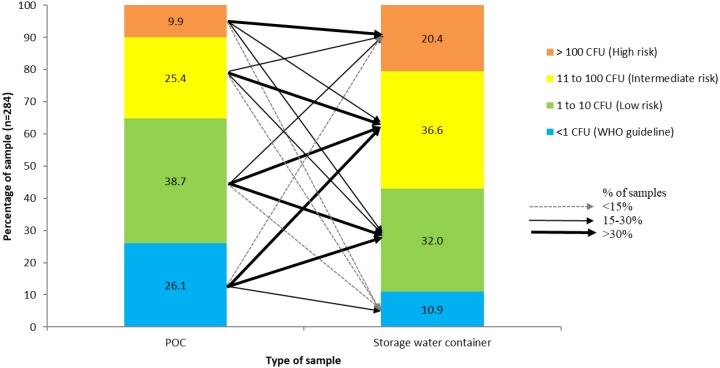
A graph of how water quality changed from POC to storage container. The values in the bar show the percentage of samples in each color category. The thickness of the arrow pointing to the second bar indicates the proportion of the samples that moved to that category.

We further analysed how the water quality changed from the POC to stored water (Figure 6), linking the POC and stored water quality (284 links in Figure 5). For example, from all POC samples that had no detectable *E. coli*, >30%, >30%, and <15% of them became low-, intermediate-, and high-risk levels in the household stored samples, respectively. By contrast, for stored water from a source that had any *E. coli* contamination (i.e., ≥1 CFU 100 mL), the stored water quality tended to remain in the same risk classification group as at the POC, i.e., at least 30% of the POC samples remained at the same risk (thickest line). 

Of the 284 stored water samples that were connected to a POC, information on HWT practices was reported by 244 households. Among these households, 41 (14%) reported at the time of the visit that they treated their drinking water. However, we did not ask about the treatment method that they used. Among these 41 respondents, the mean log_10_ *E. coli* concentration of the stored water was 0.98 CFU/100 mL (SD = 0.81) compared to 0.64 CFU/100 mL (SD = 0.86) in POC samples, indicating that HWT did not effectively improve drinking water quality at the household level. A comparison of the distribution of log_10_ *E. coli* concentrations in households that did and did not report using HWT did not reveal a statistically significant difference between these two groups (U = 1.587, *p* ≥ 0.05). 

We then performed a bivariate analysis of the factors expected to influence the microbial water quality of stored water. We found that better water quality at the POC was associated with better stored water quality (*r_s_* (284) = 0.246, *p* < 0.001). When examining possible pathways for (re)contamination at the household level, we found that the presence of livestock near the water storage was positively correlated with faecal contamination of stored drinking water (*r_s_* (200) = 0.179, *p* = 0.011). However, we did not find any significant relationship with other hygiene factors, such as how households reported cleaning the storage container (cleaning method), the presence of flies around the household, the presence of a cover on the water-storage containers, the visual cleanliness of the toilet, whether households reported using the same or a different container for transport and storage, the type of floor material, and wall material. 

From 26 triplications conducted, almost all of the samples (n = 23) had a standard deviation below three while the remaining three had the standard deviation ranging from 3 to 7, indicating that the triplication of the water samples were relatively similar to each other. 

### 3.3. Predictors of the Use of Household Water Treatment

We measured socio-economic characteristics expected to relate to the use of HWT, and the following factors demonstrated a significant association with HWT use: (1) wealth index (*r* (448) = 0.197, *p* < 0.001), (2) highest level of education completed (*r* (451) = 0.175, *p* < 0.001), (3) contact with HWT promotional activities (*X*^2^ (1) = 14.49, *p* < 0.001), (4) village (*X*^2^ (1) = 67.8, *p* < 0.001), and (5) the type of water source (*X*^2^ (2) = 23.8, *p* < 0.001). Respondents from Surkhet were also more likely to use HWT compared to other study areas (54%, Table 1). In terms of the share of households using HWT by source type, piped system (28%) and surface water (28%) users practiced HWT more often than tube well users (7%). The presence of a child in a household, whether the child had an episode of diarrhea in the past two weeks, and the time to walk to the water source were not significantly associated with the use of HWT.

We tested the association of psychosocial factors expected to relate to the perception of risk and the use of HWT. All factors were significantly and positively correlated with the use of HWT except the perception of one’s own drinking water quality (Table 2). However, among all 5 psychosocial factors, only the *perception of the risk for getting diarrhea from drinking untreated water* had a significant negative association with *E. coli* concentration (*r_s_* (481) = −0.147, *p* = 0.001). 

**Figure 7:**
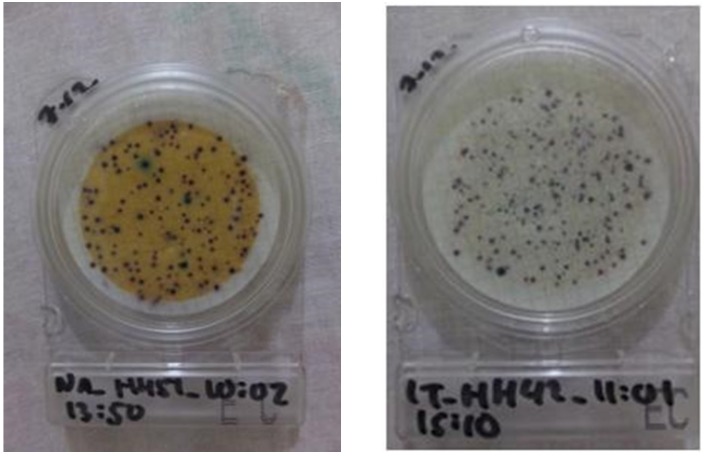
Typical water samples from Kailali (**left**) and the other four districts (**right**).

We compared the perception of risk in Kailali to other locations, since the surface water in Kailali was visibly contaminated, i.e., yellowish in color (Figure 7). Respondents in Kailali, whose majority used tube wells as their main water source, perceived poorer quality drinking water compared to other locations (*H* (4) = 44.481, *p* < 0.001), although surprisingly this perception did not lead to an increase in intent to use HWT. Instead, respondents in Surkhet had the highest intent to use HWT compared to other locations (*H* (4) = 19.365, *p* = 0.001), which was also associated with a higher percentage actually using HWT compared to other villages (Table 1). 

## 4. Discussion

This study shows that reliable water quality testing can be conducted in remote locations and evaluates microbial water quality in the hilly rural regions of Nepal. In addition, by including the flat Kailali region as one of the study districts, we could critically compare water quality and social demographics between the hilly and flat regions. This article also contributes to the global data repository of drinking water quality, especially for hilly and remote locations that are often underrepresented in such databases. 

### 4.1. Factors Related to Water Quality

Our study showed that, for most respondents, microbial water quality tended to deteriorate after collection. A decline in water quality was largely due to: (1) (re)contamination during transport and hygenic handling between water collection and consumption, and (2) the majority of respondents foregoing any form of treatment at the household level, as well as the fact that the use of HWT, i.e., self-reported use of HWT, did not positively impact the water quality. 

Most of the indicators of household hygienic conditions did not have a statistically significant relationship with stored drinking water quality. The main exception was of the presence of livestock in close proximity to the storage container, which had a weak but significant correlation with faecal bacteria concentration in stored drinking water (last paragraph in Section 3.2). A positive association between the presence of livestock and stored drinking water quality has been established by studies in Ghana, Nepal and Bangladesh, among other places [25,26,27]. We suspect that the presence of animals near the water storage is a driver of its (re)contamination, especially since structured observations revealed that 32% of households did not cover their water storage vessel, which likely increases the chances that contaminants are transferred from animal excreta to stored water.

A study in India found that the practice of keeping livestock near the water storage can reduce the positive health impact of other sanitation interventions, such as a reduction in open defecation practices [28]. Achieving open defecation free (ODF) status has been one of the main focuses of Nepal’s national government since the 2000s [29]. The situation is yet more complex in Nepal, where traditional and dominant religious culture greatly honors livestock ownership [30]. Consequently, two-thirds of the observed households in our study had livestock in close proximity to their water storage container on the household plot. This shows that socio-cultural aspects may affect the efficiency of WASH interventions in LMICs, especially where cultural practices influence water-related behaviors. WASH program implementers should think about tailored solutions for reducing rural households’ exposure to animal-feces contamination in this setting. For example, programs could promote corralling animals separately from the household’s main areas for preparing food and drinking water, ideally in combination with safe water storage methods that eliminate any contact with the surrounding environment. The latter has been found effective for improving water quality and reducing the rate of diarrhea among children in Bangladesh [31]. 

Only 41 households (from a total of 244 paired POC–stored samples, see Figure 5) reported practicing a form of HWT. However, among these households, the water quality at the household level only improved in 11 households. This finding might be due to ineffective or incorrect HWT practices, which may be the result of ineffective HWT promotion; 59% of the respondents had participated in HWT promotional activities, but among these households, only one in four actually practice HWT, and those practices largely did not improve the water quality. Meierhofer et al. [4], who conducted a study in the same region, found that the deterioration of water quality after treatment was due to inadequate ceramic filter handling and use. Studies have found that correct and consistent use of HWT can reduce the risk of water-related diseases, such as diarrhea [32,33]. Therefore, it can still be worth promoting correct and consistent use of HWT among the target groups as discussed by Daniel et al. [15]. 

This study also highlighted the fact that sanitary inspection results at the household level did not reliably predict microbial quality of stored water, as has been raised by other studies [21,34]. Instead, only one variable is significantly correlated with storage water quality (last paragraph in Section 3.2). The fact that the sanitary inspections often fail to predict actual microbial test results highlights the complexity and dynamic nature of water contamination risk factors at the household level, especially for those rural households that must transport and store their daily drinking water. These findings raise questions about the adequacy and completeness of common sanitary inspection checklists at the household for predicting actual health risks. These finding are corroborated by Robinson et al. [21], who argued that sanitary inspection forms in rural Nepal, while useful for general risk assessment and risk management of spring-fed piped water schemes, cannot replace regular water quality testing for monitoring purposes. 

There was a significant negative correlation between the number of hours water was delivered by the system each day and the log concentration of *E. coli* at the tap (Section 3.2). This finding is consistent with the study of Kumpel and Nelson [35] in India. Therefore, improving water services at the scheme-level, such as ensuring continuous tap flow, may be one of the first critical interventions for reducing the health risks due to poor drinking water quality in the study area. This recommendation is further supported by our results, which found that the microbial water quality of stored water was significantly related to the water quality at the POC. Interventions at scheme level may even be relatively easier and quicker to implement in comparison with behavioral-change interventions focused on improving the uptake and sustained use of HWT at scale [36,37]. 

As expected, the microbial water quality in Kailali, where almost all respondents used a tube well that was contaminated by arsenic [13], was relatively better compared to other locations; although the difference was not statistically significant. These findings are aligned with the study of Van Geen et al. [14] in Bangladesh, who found that high arsenic contamination significantly correlated with relatively better microbial water quality, likely due to the aquifer composition in the arsenic-affected area. 

### 4.2. Household’s Perceptions of Drinking Water Quality

In general, households’ perception of risk played an important role in increasing the likelihood of using HWT in our study area (Table 2). We also found that the water quality was relatively good at home if the respondents reported being afraid that they would get diarrhea from drinking untreated water (paragraph 2 in Section 3.3). Special attention needs to be given to the case of Kailali as the visual appearance of the water drove people’s perceptions of its quality. The yellowish color in water or surrounding the tube well comes from the presence of iron which is often associated with the arsenic contamination [38,39]. We also found that wealthier and more educated respondents were more likely to treat their water at home. This suggests that those factors may thus facilitate the behavioral changes required to perform HWT, as was also found in the context of toilet use in Africa [40]. A comprehensive behavioral study, such as using a system-level approach combining socio-economic and adapted psychological frameworks, was suggested to better understand the root causes of the behavior [15,41].

### 4.3. Study Limitation

Some limitations may impact the validity of our results. First, this study relies on a cross-sectional design and, therefore, all water-quality analyses are based on data collected at a single time point. Consequently, any temporal variability in water quality cannot be taken into account. Previous studies comparing the quality of tap and stored water in India showed high fluctuations of microbial water quality throughout the year, which would be expected in our study area [42]. Second, 24 of the 725 water samples were processed between 6–7 h after collection (as opposed to the <6 h of the rest of the samples) due to the long walking distance between the sampling location and the field laboratory. However, the exclusion of these samples would not change the main findings of our paper since the proportion was small (3% of total POC and stored samples). Third, due to time and field resource constraints, we did not perform a sanitary inspection of the water supply scheme, which limits our ability to evaluate and compare water quality risks at the POC. The WHO’s Water Safety Plan (WSP) approach recommends sanitary inspections throughout the water distribution system. Future research should analyze water quality parameters and sanitary inspection data at different points across the scheme to better understand the utility of inspection methods. Finally, the main limitation of this study is that we could not directly observe how respondents performed their HWT method in order to determine if the the overall poor water quality outcomes were explained by incorrect techniques or inconsistent practice. 

## 5. Conclusions

This study expands our knowledge of microbial quality of drinking water in remote, hilly locations with limited resources. The mobile microbial water-quality testing kit and procedure functioned reliably and is a suitable solution for conducting scientifically reliable water-quality tests in such resource-limited settings. Our approach can be replicated by stakeholders in similar locations or in unserved regions to monitor the microbial water quality and thereby inform the prioritization of a “point of intervention”. Our findings show that most households’ stored drinking water in rural hilly areas of Nepal are contaminated with fecal bacteria, mainly at a low- to intermediate-risk level. Furthermore, contamination levels were found to be higher at the household rather than at the POC level, indicating a persistent challenge with the transport, handling, and storage of drinking water. Also, the water was observed to be stored under unsanitary conditions, i.e., in containers lacking of lids and in the same vicinity as livestock. These findings suggest both a need for improving the handling and storage practices of drinking water as well as the quality of the water supplied by the local water supply schemes as a means of reducing health risks related to the drinking-water supply chain. 

## Figures and Tables

**Table 1:** Household survey respondents’ characteristics, water sources and microbial quality.

Variables	Surkhet (%)	Accham (%)	Dailekh (%)	Kailali (%)	Jajarkot (%)	Total (%)
Number of household survey respondents	93 (18)	103 (20)	113 (22)	129 (25)	74 (14)	512 (100)
*Education (total: 512 respondents)*						
No education	41 (8)	60 (12)	67 (13)	44 (9)	43 (8)	255 (50)
Primary	24 (5)	22 ( 4)	21 (4)	40 (10)	12 (2)	119 (23)
Secondary	22 (4)	16 (3)	15 (3)	29 (6)	9 (2)	91 (18)
College or higher	6 (1)	5 (1)	10 (4)	16 (3)	10 (2)	47 (9)
*Primary water sources (512 respondents)*						
Tap water (either in own house or community tap)	57 (11)	98 (19)	89 (17)	0 (0)	60 (12)	304 (59)
Tube well	0 (0)	0 (0)	0 (0)	127	0 (0)	127 (25)
Rain-water harvesting	0 (0)	0 (0)	10 (2)	1 (0)	1 (0)	12 (2)
Surface water (e.g., open source, river)	36 (7)	5 (1)	14 (3)	1 (0)	13 (3)	69 (13)
*Ever received promotional material on household water treatment (HWT, 512 respondents)*						
Yes	71 (14)	56 (11)	65 (13)	79 (15)	32 (6)	303 (59)
*Presence of children under 5 years old (512 respondents)*						
Yes	38 (7)	58 (11)	63 (12)	61 (12)	45 (9)	265 (52)
*Use HWT (451 respondents)*						
Yes	44 (10)	19 (4)	18 (4)	8 (2)	10 (2)	99 (22)
Total number of stored-water samples	90 (18)	101 (20)	112 (22)	129 (25)	74 (15)	506 (100)
Mean log_10_ *E. coli* colony-forming units (CFU)/100 mL (SD)	1.14 (0.83)	1.12 (0.89)	1.23 (0.80)	1.27 (0.82	0.94 (0.83)	1.16 (0.84)
Total number of POC samples	8 (5)	25 (15)	24 (14)	65 (39)	45 (27)	167 (100)
Mean log_10_ *E. coli* CFU/100 mL (SD)	0.70 (0.97)	0.93 (0.84)	0.58 (0.92)	0.44 (0.73)	0.52 (0.97)	0.57 (0.86)

**Table 2:** Bivariate associations between psychosocial factors and the use of HWT.

Psychosocial Factors	Answer (the Lowest and the Highest Criteria)	Mean (SD)	r (n) ^a^
Perception of their own water quality	1 = Very good, 5 = Very bad	2.41 (0.81)	0.03 (450)
Perception of the safety of drinking directly from water source without treatment	1 = Very safe, 5 = Very risky	3.06 (1.17)	0.27 (436) **
Perception of the risk of getting diarrhea if drinking untreated water	1 = Very low, 5 = Very high	2.49 (1.06)	0.18 (434) **
Perception about whether HWT can prevent diarrhea	1 = Not certain, 5 = Very certain	2.99 (0.92)	0.37 (451) **
Knowledge on different methods of HWT	0 = Cannot explain any HWT methods, 4 = Can explain more than 3 methods	2.01 (1.20)	0.37 (450) **

^a^ Pearson correlation, ** *p* < 0.01.
